# Provider and Manager Perspectives on the Use of an Integrated Clinical Pathway for Community-Dwelling Older Adults: A Qualitative Case Study

**DOI:** 10.5334/ijic.5965

**Published:** 2022-01-13

**Authors:** Paul Wankah, Isabelle Gaboury, Louise Belzile, Mylaine Breton

**Affiliations:** 1 Université de Sherbrooke, CA

**Keywords:** integrated care pathways, older adults, evidence-based clinical tools, home care

## Abstract

**Background:**

Integrated care pathways (ICPs) could improve the organisation and delivery of care for community dwelling older adults. An ICP was developed and implemented in Québec to support home care processes. This study explores the perspectives of home care staff on the use of an ICP to support the organisation and delivery of health and social care to community-dwelling older adults with complex needs.

**Theory and Methods:**

A case study based on eleven semi-structured interviews and analysis of documents was carried out in an urban home care unit. The Normalization Process Theory was used for mixed thematic analysis.

**Results:**

While its capacity to store data and enhance interprofessional information exchange was appreciated by home care staff, the broad scope, and automated features of the ICP tool were often problematic. Concerns about increased provider workloads, disruption to provider-client relationships during clinical encounters, and difficulties engaging clients in decision-making were main obstacles in the use of the ICP.

**Conclusion:**

Given the importance of ICPs in advancing clinical integration, it is critical to continuously adjust their design to align with providers’ realities in order to optimize their potential in real life contexts.

## Introduction

Demographic and epidemiological trends of industrialised countries over the last few decades reveal increasing proportions of community-dwelling older adults living with complex health and social needs [1]. These persistent trends exert pressure on the organisation and delivery of care for people living with complex sociosanitary needs, for they require a comprehensive range of seamless services from various providers operating in different health and social care organisations.

Home care ensures the provision of health and social services that enable people living with complex sociosanitary conditions to remain at home [2,3]. Home care services are offered by interprofessional teams of providers and involve a wide array of services, such as medication management, help with activities of daily living, caregiver respite, and health education [3,4]. Home care providers establish referral processes to ensure continuity with specialized services, and coordinate follow-up mechanisms to tailor services to the needs and preferences of their clients [5,6]. Despite overall improvements in the organisation of home care services, several studies have reported wide variations in the type and quality of services offered in home care programs, with potential negative impacts on people living with complex needs [3,7,8]. To address these variations, Integrated Clinical Pathways (ICPs) have emerged in many countries as organisational strategies to standardize and improve the effectiveness of home care services [9,10,11].

ICPs consist of a model that “explicitly states the goals and key elements of care based on Evidence Based Medicine guidelines, best practice and patient expectations by facilitating communication, coordinating roles and sequencing the activities of the multidisciplinary care team, patients and their relatives; by documenting, monitoring and evaluating variances; and by providing the necessary resources and outcomes” ([12] p.553). Recent literature reports important issues related to the uptake and usage of ICPs in organisations. These include challenges in using information systems to disseminate clinical tools and to support clinical and administrative decision making while allowing for collective action [13]; challenges in building effective multidisciplinary clinical teams to carry out a sequential, complementary and interdependent range of activities that are coherently aligned to achieve common goals [14]; and challenges in strengthening the evidence for clinical and administrative processes used in ICPs [15]. Important knowledge gaps remain on how ICPs are used to support the daily work of home care providers [16]. The general objective of this study is to explore the perspectives of home care staff on the use of an ICP to support their efforts to organise and deliver health and social care to community-dwelling older adults with complex needs.

## Study context

In the Quebec healthcare system, 95 local health networks [17] are responsible for organising, managing, and delivering health and social services to their local populations [17,18]. Each network administers a Support Program for the Autonomy of Seniors (SAPA) that includes long-term care, home care and community-based resources to meet the needs of older adults. Home care consists of interprofessional teams of providers (nurses, social workers, occupational therapists, physiotherapists, etc.) that offer a broad range of services, including nursing care, healthy lifestyle and falls prevention, caregiver respite and nutrition services [19]. One strategy used to improve the effectiveness and efficiency of services consists in attributing case managers to complex clients to enhance care coordination across organisational and professional boundaries [20].

In 2007, an ICP named the *Outil de Cheminement Clinique Informatisé* (OCCI) was developed to enhance clinical and administrative practices in home care [9]. The OCCI is a clinical management system for community dwelling older adults that was designed to support five phases of clinical activities [9]. These phases of clinical activities are high-level representations of clinical processes that constitute the continuum of care for older adults. Specifically, *phase one* consists of a comprehensive assessment of needs, risk and protective factors of community-dwelling older adults and their caregivers; *phase two* consists of collecting and summarising patients data, as well as identifying patient care goals; *phase three* consists of care planning from the values and priorities of clients; *phase four* consists of coordinating the delivery and follow-up of services; and *phase five* consists of analysing outcomes, identifying variations, and planning reviews and adjustments of services [9]. Basically, the OCCI was designed to facilitate clinical activities by providing a structure for daily work routines.

The OCCI has been progressively and partially implemented in home care across the province [16]. In 2012, the first two phases of OCCI were introduced into clinical information systems used in local health networks [16]. Then in 2014, the first three phases of OCCI were incorporated in a ministerial computerized clinical management system [16]. At its current level of implementation, the OCCI can only support *comprehensive needs assessment, data collection and storage*, and the *care planning for clients and their families* [16].

This study focuses on the **needs assessment** and **care planning** work of home care providers. Despite the potential for OCCI to enhance the speed, effectiveness and comprehensiveness of needs assessment and care planning, there is limited understanding of how frontline providers use the OCCI in their daily work routines. To the best of our knowledge, no previous study has examined the experience of providers with these tools, and we consider that understanding how OCCIs are used in real-life contexts may help design strategies to improve needs assessment and planning in home care. The two specific objectives of this study are:

1. To describe the needs assessment and care planning work of home care providers.
2. To identify and examine perceived facilitators and barriers to the use of OCCI in the daily work routines of home care providers.

## Conceptual framework

The Normalization Process Theory (NPT) [21,22] is a conceptual framework used in implementation science to examine how various complex healthcare interventions support clinical activities in different contexts [23,24,25,26]. We adopted the NPT in our analysis as it focuses on understanding “the processes through which a practice or practices become, (or do not become) routinely incorporated in everyday work of individuals and groups” ([22] p.2).

The NPT suggests that uptake and usage of an intervention depend on the individual or collective efforts of organisational actors to implement them [21]. It is therefore necessary to “look at what people actually do and how they work” [21]. Furthermore, the NPT suggests four constructs – **coherence** (the sensemaking work of actors), **cognitive participation** (the relationship work of actors), **collective action** (the enacting work of actors) and **reflexive monitoring** (the appraisal work of actors) [21,23,24] – as four different kinds of work that contribute to establishing a new practice in an organisational setting.

This study focused on the “collective action” construct of the NPT because it explores collective efforts of actors in the routine incorporation of innovative practices in social contexts or “how the work gets done” ([21] p. 549). This construct also suggests that the collective efforts of actors is shaped by environmental factors that enhance or inhibit their ability to enact new practices. Furthermore, we were interested in exploring factors that influenced the routine enactment of a new practice which is not captured by the other constructs of the framework.

## Methods

### Study design

This research project used an exploratory case study design [27] to explore how an innovative Integrated Care Pathway – the OCCI – was used to support routine clinical activities of needs assessment and service planning for community-dwelling older adults as perceived by home care staff. The case is defined as the process of using OCCI by home care staff in their routine activities [27,28].

### Setting and study participants

The study was carried out in one home care unit of an urban Local Health Network in Quebec, Canada. The site had implemented the OCCI one year before the beginning of this study but had not been involved in the pilot phase of its development. This case site was purposefully chosen [29] for two reasons,: i) because it allowed us to capture the everyday work conditions of a typical home care unit in Quebec, and ii) because it enabled us to explore the interactions of staff who used the OCCI in their routine clinical practices to deliver care to clients.

Study participants were five providers and six middle managers involved in the continuum of care for older adults in the home care unit (***Table 1***). Participants were conveniently selected [30] based on their function in the home care unit, to provide a mix of informants with valuable insight into how OCCI supported clinical practice. Providers were healthcare and social services professionals involved in clinical decision-making roles and the provision of direct care. Managers were home care unit staff providing administrative and clinical support, training, and resources to frontline providers.

**Table 1:** study participants.


TYPE OF PARTICIPANT	ROLES IN HOME CARE	NUMBER OF PARTICIPANTS

Providers	Nurse (N)	2

	Social worker (SW)	1

	Occupational therapist (OT)	1

	Nutritionist (Nu)	1

Managers	Head of unit (HU)	1

	Coordinator of occupational therapists (C)	1

	Coordinator of nursing (C)	2

	Coordinator of social workers (C)	1

	RSIPA* solutions trainer (T)	1

**Total number of participants**		**11**


### Data collection

Data sources for the case study [27] included semi-structured interviews [31], and document analysis [32].

#### Semi-structured interviews

Semi-structured interviews focused on generating an account of how home care providers used the OCCI in needs assessment and care planning. Eleven semi-structured, face-to-face interviews were conducted between June and September 2019. The interview guide prompted participants to describe the usage of OCCIs in the clinical workflow of providing home care, the various instruments used to assess client needs and plan care, the roles of clinical staff in organising and delivering care, and difficulties encountered with the OCCI during routine clinical work. Interviews, lasting an average 60 minutes, were audio recorded and transcribed.

#### Document analysis

Relevant documents were reviewed to understand the content of the OCCI, the home care context in which the OCCI was used, and the clinical practices meant to be supported by the OCCI. These included official documents such as practice guidelines for home support.

### Data analysis

Qualitative data analysis was facilitated by the NVivo 11 software [33]. This study used a deductive and inductive thematic analysis approach [34].

A codebook was structured around the collective action construct of the NPT [21] to organise and condense data from interviews and documents. Two researchers (PW and LB) reduced the qualitative data. Data was then classified into emergent sub-categories that represented key stages in the flow of clinical activities that occurred while using the OCCI to support needs assessment and planning of services in the home care unit.

**Figure 1:**
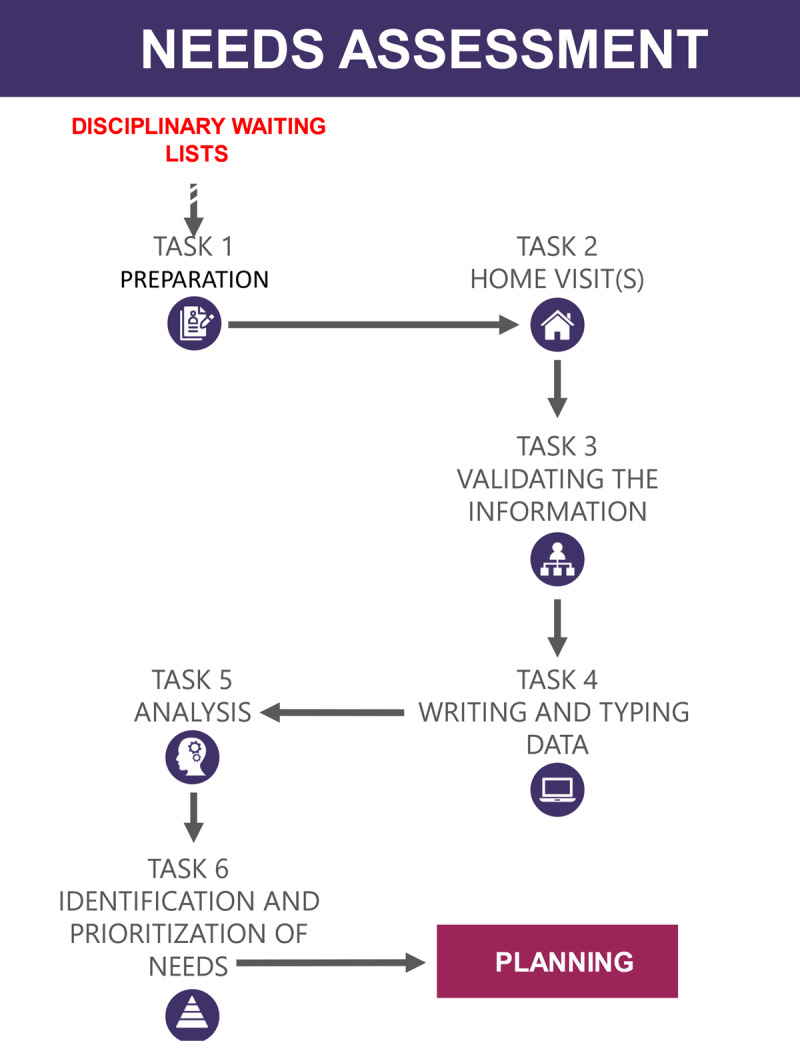
Tasks involved in needs assessment.

**Figure 2:**
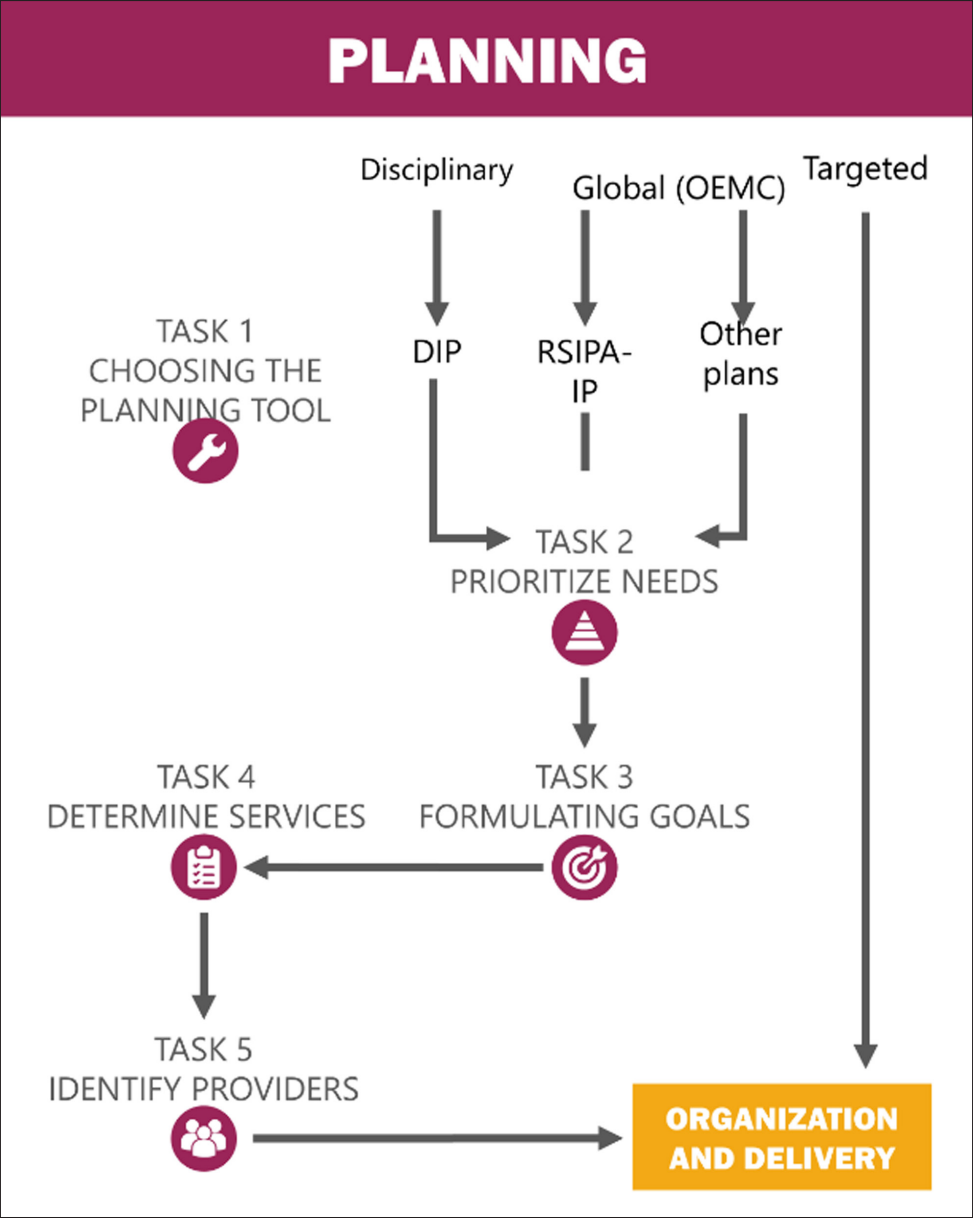
Tasks involved in care planning.

We presented a preliminary outcome mapping (see ***Figures 1*** and ***2***) based on this analysis to an advisory committee composed of two managers from the Ministry of health and social services, three managers from the local health area, and three researchers. Feedback from these informed respondents enabled us to validate and adjust our interpretation of providers’ experiences, and develop the narrative presented in the results section below.

### Ethics

This study was approved by the local Ethics Committee: reference number CE-HCLM-17-064.

## Findings

### Needs assessment

The comprehensive assessment of client needs is a crucial stage in home care practices supported by OCCIs. It involves a range of clinical activities to collect and systematize patient data and translate these into needs. ***Figure 1*** presents the six types of tasks of needs assessment as reported by home care staff.

#### Preparation

All respondents recognised that some sort of preparation was necessary before meeting clients in their homes for direct needs assessment. Respondents identified three types of activities prior to their initial patient encounter. First, providers consulted patient case notes and other documents to review issues related to the client’s clinical conditions and select evaluation tools to use during the home visit. Second, they held informal discussions with other providers who could help them understand the client’s condition. Most respondents pointed out that OCCIs had an important role in shaping conversations between providers by creating a common language and focussing discussions on the dimensions addressed in the tool. The OCCI was also perceived as facilitating effective interprofessional collaboration and communication.

“I think that OCCIs have a role in interprofessional collaboration by making it possible to collect data that is also useful for other providers […]. I completed [the evaluation tool] for the treatment team of the other [organisation], telling myself they are going to have the real portrait of [the client].” (Social Worker)

The final aspect of preparation consisted of initiating contact with the client to schedule a home visit.

#### Home visits

The mainstay of needs assessment occurred in the client’s home. Depending on the complexity of the case or the frailty of the client, the provider could schedule one or more home visits. This task involved data collection, such as patient information, clinical symptoms, or psychological and social needs. Three types of needs assessment tool were used to collect clients’ clinical data: i) the Multiclientèle Evaluation Tool that was integrated into the OCCI and used for comprehensive needs evaluation; ii) the disciplinary evaluation tools (e.g. specific to nursing or occupational therapy) as required by their professional orders; and iii) targeted evaluation tools for specific issues or pathologies (e.g. cognitive impairment, nutritional status, caregiver burden, mobility, etc.).

Most providers suggested that the OCCI facilitated data collection during home visits for two reasons; i) because all the elements of the comprehensive evaluation tool were integrated as a list in the OCCI, and ii) because some data from the OCCI assessment supplemented the disciplinary evaluation tools.

“I start with the OCCI and complete an occupational therapy assessment afterwards. I’m more comfortable proceeding like that. I’m sure I haven’t missed anything. I can then refer to the OCCI during my occupational therapy assessment, rather than repeating everything […]. It’s precisely this type of case where there’s very little information. The OCCI allows for more global data collection, which anchors the rest.” (Occupational Therapist)

#### Validation of information

The next task for home care providers was to validate the data collected from clients. Information stored in the OCCI was verified and updated by i) contacting the client’s caregiver/family to clarify certain points or obtain additional information; ii) contacting other healthcare professionals involved in the client’s care; and iii) consulting the client’s medical record for supplementary information.

Providers pointed out the value of being able to store clients’ clinical information on the OCCI. This gave them the flexibility to validate client information after the home visit, and to see the evolution of the client’s situation over time.

#### Writing up and entering data

The next task consisted of documenting the client’s data. Providers could do this in different ways – entering patient data directly onto computerized information systems (which was the practice promoted by the home care unit), writing client data on paper tools, and completing client progress notes to supplement the needs assessment.

The OCCI could potentially facilitate entering data directly onto the computerized information system while in the client’s home. However, few providers were comfortable with this procedure. Some reported that typing information during home visits disrupted the flow of conversation with clients and their ability to observe non-verbal client communication. Hence, most preferred to take brief notes during the patient encounter and complete the OCCI later. Although providers appreciated the storage function of OCCI, they found that relational work with clients suffered when they had to complete computerised notes in the client’s presence.

“Is it my discomfort or their discomfort? I haven’t met anyone who outright refused that I take out my computer. In fact, I don’t take it out, even though that’s an obligation with the OCCI […]. Some clients tell me that the doctor doesn’t look at them, that he looks at the computer. I imagine that applies to me too! We work with humans; we don’t work with machines. It makes me think “if I want to establish a bond of trust, I have to be able to speak to the person as if I was having afternoon tea with them.” (Nutritionist)

#### Analysis

Providers mentioned two distinct approaches to analyzing client data: automated analysis and manual analysis. The main contribution of the OCCI was its capacity for automatic analysis of client data, which was appreciated by most providers. First, OCCI generated an initial list of elements identified as problematic and proposed interpretations that might focus attention on issues such as elder abuse, nutritional risk, or caregiver burden. The provider then used their clinical judgement to decide whether to retain and follow-up on the automated suggestions. In addition, the OCCI automatically generated a profile of the client at the end of the assessment. OCCI also grouped data to create a summary assessment and results could be directly transferred to the intervention plan. Finally, certain aspects of a client’s data had to be manually analysed, such as clinical data that did not fit a typical profile, or that were not used in determining a client’s loss of autonomy.

#### Identification and prioritization of needs

The last task required client input to identify and prioritize their needs and prepare for the care planning stage. Several respondents acknowledged that OCCIs made it possible to identify for whom the situation was problematic (the client, caregiver or provider), and suggested priority problems to be addressed. Nonetheless, providers pointed out that this function was rarely used as planned because this step came at the end of needs assessment. Given that most providers completed and validated client information after the home visit, the needs prioritization task was not often accomplished in the presence of the client. Furthermore, older adults were often exhausted by the time the comprehensive evaluation of their needs was completed.

“…what I try to do is distinguish what the client values, what the informal caregiver values, and what the provider values. Then to indicate who wants what, what the client is willing to work on. That’s what helps to prioritize … but I’m not sure it’s really complete. Clients are often exhausted by then.” (Social Worker)

### Factors that influenced the use of OCCI in needs assessment

Respondents drew attention to factors that positively or negatively influenced the way the needs assessment work was accomplished with the OCCI (***Table 2***). Difficulties engaging clients in prioritizing their needs and increased workload on providers were the main obstacles. The potential to improve work processes by sequencing clinical tools was the main facilitator. There were mixed views on the OCCI’s contribution to clinical judgement.

**Table 2:** Factors influencing the usage of the OCCI in needs assessment and care planning.


FACILITATORS	OBSTACLES

• The OCCI facilitated interprofessional communication around dimensions of client health care needs addressed by the tool. • The OCCI facilitated data collection and storage in home care setting • Automated feature of the OCCI could enhance the efficiency and effectiveness of needs assessment • The OCCI facilitated sharing of care plans to several providers	• The OCCI disrupted provider-client information flow during clinical encounters. • Routine usage of the OCCI increased the workload of providers. • The automated feature of the OCCI influenced the capacity for clinical judgement of providers • Difficulties to involve clients in determining care goals with the OCCI. • Several important resources and services were not included in the computerized list of the care plan


Providers felt that several important needs assessment tasks were still being performed without the client’s contribution. Rather, they were done *for* the client. In fact, the prioritization of client needs was not done during the patient encounter, but rather back in the office when entering data into the OCCI.

Routine use of the OCCI seemed to complexify and increase the work burden of home care providers. Providers described completing the OCCI as a very long process and it did not exempt them from the obligation to complete other disciplinary (nursing, social work, etc.) and targeted assessment tools. OCCI was designed to evaluate global client health and social care needs, not specific disorders. Some providers pointed out that limited access to information systems reserved for certain professionals complicated preparation and validation tasks. Other providers mentioned duplication of information between clinical tools and between information systems.

Providers pointed out that the OCCI could improve their work processes if they sequenced completion of different needs assessment tools. They suggested that using OCCI first for a global assessment of client needs, followed by disciplinary evaluation tools and then targeted evaluations could improve efficiency. Once the global needs assessment was completed, the disciplinary assessment took less time.

Respondents had mixed views on the OCCI’s influence on their capacity to exercise clinical judgement. On the one hand, the OCCI enhanced needs assessment by providing a multidisciplinary clinical tool and an IT platform that allowed automated analysis of client needs. This could potentially increase efficiency and effectiveness in identifying client needs and reduce the risk of forgetting some client information. On the other hand, providers were wary of the automated analysis. None of the respondents (even the trainers) used the feature to transfer automated OCCI analysis to their care plan, preferring to rely on their professional judgement.

“When you click “Generate a plan,” I think it’s all coming up. I think [the computerized tool] thinks for us. “Let me have some clinical judgement, please! I’m going to do it, my care plan!” Probably my resistance started from that and I never wanted to do it because I thought: I’m going to set my own goals. So, I’ve never used the “Generate a plan” feature.” (Social Worker)

### Care planning

Care planning was the next stage in home care to be partially supported by the OCCI. Briefly, this stage involved a range of clinical activities leading to the development of clinical care plans that addressed the client’s needs. ***Figure 2*** presents tasks involved in care planning as perceived by home care staff.

#### Choosing the planning tool

Respondents stressed the importance of choosing the most appropriate tool for planning care. Home care providers developed two main types of care plan: i) the *disciplinary care plan* that aimed to address client issues linked to a specific disciplinary field like nursing, nutrition, or occupational therapy, and ii) the *interprofessional care plan* that aimed to address complex health and social issues that required coordinated efforts of several providers. Priority needs related to a specific discipline were followed up with a disciplinary care plan, while priority needs requiring care coordination were followed up with an interprofessional care plan. The OCCI did not effectively contribute to the task of choosing the appropriate type of care plan.

#### Prioritizing needs addressed in the care plan

The OCCI allowed providers to transfer client needs identified during the needs assessment stage to the disciplinary care plan.

#### Developing goals

Although care plans were supposed to be tailored to client preferences, most providers acknowledged that they rarely engaged clients in this stage and often formulated general rather than personalized goals in order to work faster.

“Setting goals is very complicated. I use the same ones pretty much all the time. So the care plan is general. There are so many options, but we don’t want to search for 15 minutes and end up with a six-page care plan to detail everything.” (Social Worker)

#### Determining services

The OCCIs provided drop-down lists that were intended to facilitate the identification of resources. In practice, respondents pointed out that these lists made the job more difficult and that they spent a lot of time looking for the right way to phrase keywords. It was also impossible to list all the resources (especially if they were less conventional) that could be mobilized to meet a client’s specific need. Providers reported that they preferred to select or write very general services in the OCCI and indicate the most relevant services in the comments area.

“In the past, we used to make care plans in the form of beautiful tables in which we wrote the problem, the objective, the means of intervention, the timetable. So that’s something we were already doing. The OCCI have not revolutionized anything, it is the container that has changed. And now for the means of intervention, well, we can no longer write what we want.” (Coordinator)

#### Identifying service providers to meet the client’s goals

The OCCI provided a drop-down list of additional health and social care providers, enabling the provider developing the care plan to select and add other providers who could add value to their client’s care.

### Factors that influenced the use of OCCI in planning services

Respondents drew attention to factors that negatively influenced the way care-planning work was accomplished with the OCCI (***Table 2***). These included difficulties in using OCCI to tailor services to the preferences of clients and identify services, and concerns about the clinical utility of care plans.

Home care staff pointed out that care plans should ideally contain concise information that allowed any provider to know what was being done to meet the needs of a client, the other providers involved in the continuum of services, the type of services being provided and how frequently they were being provided. In practice, home care staff were sceptical of the way care plans were developed because it seemed to fall short of tailoring services to the preferences of clients. It was often difficult to involve clients in determining goals or identifying services for their care plans. Furthermore, care plans were often drawn up according to the availability of resources instead of client preferences. These problems were not new but use of the OCCI in care planning did not begin to resolve them.

Several respondents expressed concerns about the clinical utility of care plans following comprehensive assessments. They did not think the computerized care plan facilitated planning activities, because information was still scattered across various documents (including the OCCI) and information systems. The goals of care plans were perceived as being limited in number, general and not very personalized, and providers considered them unreliable and not terribly useful for organising services or reporting what was done for the client.

## Discussion

A major goal of integrating health and social services is to improve clinical integration, which directly affects clients living with complex socio-sanitary needs [35,36]. Nonetheless, several studies show that difficulties in achieving clinical integration across settings often hinder integration efforts [37,38]. This case study drew on Normalised Process Theory to examine how frontline providers used a partially implemented integrated care pathway – the OCCI [9] – to carry out their needs assessment and care planning work. This theoretical approach allowed us to identify opportunities to improve the routine usage of clinical tools designed to enhance integrative clinical practices.

ICPs are evidence-based tools designed to enhance clinical practices [16]. Although these tools are known to support interprofessional collaboration by ensuring effective exchange of relevant client data, enabling comprehensive needs assessment, or supporting clinical decision-making [39] there has been little scholarly attention paid to the extra workload they create for providers. Comprehensive clinical tools may contain multiple indicators that cover different aspects of a client’s condition. Although the comprehensive nature of the tool provides a base for improving clinical practice, our findings show that this is often associated with longer clinical processes that increase provider workload and client fatigue. Given that increased provider workload is associated with reduced quality of care in the primary care setting [40], it is important to find a balance when adopting ICPs. Evidence from another setting [41] suggests that stakeholders should pay attention to the real possibility of increased workloads when designing and implementing integrated care initiatives.

Client-centredness is a core principle of integrated care practice that emphasizes the need to tailor services to the preferences, values and priorities of clients [42]. Although multiple studies have suggested macro, meso and micro-level strategies to foster client engagement in clinical decision-making [43,44], our findings concur with other studies showing that effective client engagement remains problematic [45,46]. In our study, the later stages of needs assessment and care planning were carried out in the offices of providers, not with clients in their homes. Providers also pointed to the complexity of the OCCI platform as an obstacle to customising client care goals. This finding should encourage organisational leaders to examine context-specific challenges to effectively capturing the preferences of clients during a clinical encounter supported by an ICP. Continuous quality improvement approaches that involve patients may help adjust organisational processes to this end [43,47]. Other studies recommend extended training in monitoring, feedback and practice support systems to improve patient engagement [45,46,48] and improve provider skills in the use of technologies [49,50].

Evidence-based clinical tools are often supported by IT systems in integrated care settings [51]. While the potential for IT to improve healthcare processes is widely recognised [51], our findings show that they also entail challenges. For instance, our respondents appreciated the capacity of the OCCI to store patient data and facilitate interprofessional communication but deplored having to enter patient data directly into the computer during home visits as it compromised the provider-client relationship. Other studies addressing the impact of IT on provider-patient relationships suggest including relationship-centred care in informatics research [52]. Virtual assistants and portable devices that can record clinical interactions and convert data to text have also been suggested as ways to improve the integration of IT into the provider-client encounter [53].

Automated features of ICPs were designed to quickly process clinical data and support clinical-decision-making [9,16]. While some providers appreciated the capacity for automated functions to reduce the duration of their work processes, others were concerned that automated analysis undermined their clinical judgement. In other words, they were concerned that the “machine” was trying to replace them. This paradox highlights debates on the impact of computerized clinical decision support systems on professional autonomy in the health sector [54]. While ethical and practical considerations on the utility of computerized clinical decision support systems will continue to evolve, ICP designers should recognise and address the concerns of providers who value clinical judgement as part of their work. Automated analysis is perhaps best added as an optional feature in the design of ICPs.

This study focused on understanding experiences of home care staff that used OCCIs in their clinical activities and did not include the perspectives of clients. Future studies might seek to understand the experience of home care clients assessed with the OCCI to gain a fuller picture of the benefits and shortcomings of this tool. Finally, this study only examined the perspectives of 11 respondents. While this limited number of participants was adequate to attain data saturation [27] in our study, future studies may consider other contexts or multiple cases to enhance the credibility of their findings.

## Conclusion

ICPs are important clinical tools to support the daily work processes of home care staff working with community-based clients living with complex needs. This in-depth theory driven examination of the use of the OCCI by home care providers in Québec reveals challenges and opportunities to improve clinical work processes. Providers appreciated the OCCI’s storage function and capacity to improve interprofessional communication but deplored its complex platforms and the difficulties it created for engaging patients. Given the importance of ICPs to advancing clinical integration, it is important to continuously adjust the design of these tools to the realities of stakeholders.

## Additional File

The additional file for this article can be found as follows:

10.5334/ijic.5965.s1 Annexe A. Interview guide.
