# Supplementary material for: Provider and Manager Perspectives on the Use of an Integrated Clinical Pathway for Community-Dwelling Older Adults: A Qualitative Case Study
Source: Int J Integr Care. 2022 Jan 13;22(1):1. doi: 10.5334/ijic.5965 (PMC8782094; doi:10.5334/ijic.5965)
Supplement: Annexe A. — Interview guide. [file ijic-22-1-5965-s1.pdf]

Research project: *Optimization of clinical processes for needs assessment and care planning of home care services for seniors*

ANNEX A:
SEMI-STRUCTURED INTERVIEW GUIDE

Instructions:

We will discuss your routine practice and how you use clinical tools for needs assessment and care planning for seniors. The purpose of this interview is not to evaluate your individual performance, but to understand what you do, the constraints you face, and the strategies you deploy to deliver care to your clients.

Socio-demographic data

1. What is your disciplinary background? Your roles and functions within SAPA? How many years have you been working in this program?
2. What clinical tools do you use regularly in your work?

PART A: INFORMATION ON THE USES OF CLINICAL TOOLS (35 minutes)

**THEME 1: NEEDS ASSEMENT**

3. What is the place of needs assessment tools in your routine work?
4. Did the introduction of OCCIs changed the needs assessment activities as compared to the OEMC?

    Probing cues
    - How?
    - Since when?
    - Do you use the information that is available in addition to the enhanced OEMC (which are the clinical pathway tools)?
5. Do you think that OCCIs add value to clinical activities? Why?

    Probing cues
    - Do you think that the use of OCCI supports your professional judgment or does it hinder it?
    - Do you use OCCIs to complement other clinical tools (e.g. a disciplinary tool or the intervention plan)? Or vice versa?
6. What are the instructions/guidelines for using OCCIs? How do you understand them?

    Probing cues
    - What was presented in the training?
    - What does your organization require you to do?
7. What are the steps or tools that you need to complete, in addition to the OCCIs to complete your needs assessment? (Plot the continuum for different cases).
8. Do you do any other assessments?

    Probing cues

- If so, which ones (e.g. social functioning, in case of incapacity, disciplinary, etc.)?
- Under what circumstances?
- How often?
- Do they have links with the OCCIs?

9. Are there any particular challenges in assessing the needs of the senior with the tools you have? If so, which ones?

**THEME 2 CARE PLANNING**

10. What place do intervention plans occupy in your work?
11. Do you think IP to are useful in your interventions? What for?

    Probing cues
    - How do you choose the issues that will be included in the intervention plan?
    - When do you consult the IP you make?
    - When and how do you change them?

12. What are the user guidelines for individualized service and service allocation plans (PSIAS)that are found in the RSIPA electronic platform?

    Probing cues
    - What was presented in the training?
    - What does your organization require you to do?

13. What steps or tools do you need, in addition to PSIAS to complete your care planning? (Return to the continuum for different cases).

    Probing cues
    - Do you make connections between OCCIs and your PSIAS?
    - Are you familiar with the functions that allow data to be transferred from OCCIs to PSIAS? Do you use them?

14. Would you rather use another tool instead of the PSIAS? Why?

    Probing cues
    - Do you complement other types of planning tools?
        - If so, which ones (e.g.laptops)?
        - Under what circumstances?
        - How often?
        - Do they have links to THE PSIAS?
    - Who participates in the realization of the PSIAS? (User, caregiver, other stakeholders, doctor, etc.) ? Explain the contribution and role of professionals at each stage.

15. Do the development of PI-PSIAS present any particular challenges?

    Probing cues
    - How do you manage to involve the user?
    - Can you incorporate preventive activities into your IP?

16. How do you know if the services you are putting in place meet the needs and expectations of users and their caregivers?
17. Do you record somewhere the required services that are not provided to your clients?

THEME 3 THE RSIPA PLATFORM AND USE OF TECHNOLOGIES

18. How do you use the RSIPA platform.
    Probing cues
    - What modules do you use?
    - Has the computerization of clinical tools changed anything in your work? If so, how?
    - What improvements would you like to see to optimize the platform?
19. Would you say you're comfortable with technology in general? With the use of the computer?

THEME 4 GAP BETWEEN PRESCRIBED AND ACTUAL USES

20. Do you consider that you are responding exactly to the request (of your organization or your professional order) in the way you use disciplinary and interdisciplinary clinical tools?
    Probing cues
    - For what reasons do you do this?
    - What is your capabilty to adpat the usage of clinical tools?
    - Are there any particular elements in your practice context that explain or justify these ways of doing things? If so, which ones?
21. Do you have your own way of working with each of the tools?
    Probing cues
    - Do you complete the tools in an established order/sequence?
    - Why do you complete them in this order?

22. If you had a choice, to what extent would you use:
    - The clinical tools contained in the RSIPA?
    - Your disciplinary tools?
    - Other tools?
23. Do you value current accountability?
    Probing cues
    - Do you think the data that is used for accountability could be useful to you from a clinical point of view?
    - What data would help you better understand the relevance of your interventions or plan your work?
    - What do your managers and the MSSS do with this data you collect?
24. What do you need to work better?
    Probing cues
    - Are there certain tasks or tools that seem superfluous or useless to you?
    - What makes your workplace special?

- On what occasions do you have the opportunity to discuss difficult cases? Do you have the power to adjust the criteria for awarding services?
- What is your main wish to optimize the evaluation and planning processes?

25. How do you see yourself in the next 10 years? Will you still be working at SAD? What for?

PART B: NARRATIVE EXPLANATION OF THE PRACTICE (15 minutes)

Instructions for carrying out the second part of the interview

- To complete this part of the interview, we ask you to explain an intervention that you have made in one of the files that you have in your case load.
- Choose
  - The most recent case possible
  - In which you have completed an assessment and an intervention plan.

Nominal information on the case that will be reported will not be noted.

1. Describe this situation to me.

Benchmarks for reminders

- Context of the request (referent, living environment, purpose of the request, etc.)
- Tell me how you collected and recorded the information.
- What were the main problems?
- Grey areas or questions?
- What difficulties (technical, relational, communicational, etc.) have you encountered?
- Were you surprised by the Iso-SMAF profile score?
- How does the Iso-SMAF profile influence the course of your interventions?
- What type of intervention plan have you written?
- Clinical reasoning: Did you have different assumptions about:
  - The problems/needs of this person?
  - The solutions to be proposed to meet the identified needs?
  - How did you formulate them?
  - How did you choose the best ones?

2. Does this particular case represent the way you generally proceed to complete needs assessment and a care plan?

Closing the interview

Do you have anything to add to enhance our understanding of the continuum of evaluation and planning activities?

THANK YOU!
